# CRISPR-Mediated In Situ Introduction or Integration of *F9-Padua* in Human iPSCs for Gene Therapy of Hemophilia B

**DOI:** 10.3390/ijms24109013

**Published:** 2023-05-19

**Authors:** Qiyu Tang, Zhiqing Hu, Junya Zhao, Tao Zhou, Shuqing Tang, Peiyun Wang, Rou Xiao, Yan Chen, Lingqian Wu, Miaojin Zhou, Desheng Liang

**Affiliations:** Center for Medical Genetics, School of Life Sciences, Central South University, Changsha 410078, China

**Keywords:** hemophilia, CRISPR/Cas9, iPSCs, FIX-Padua, *F9-Padua* mutation, gene therapy

## Abstract

Hemophilia B (HB) is an X-linked recessive disease caused by *F9* gene mutation and functional coagulation factor IX (FIX) deficiency. Patients suffer from chronic arthritis and death threats owing to excessive bleeding. Compared with traditional treatments, gene therapy for HB has obvious advantages, especially when the hyperactive FIX mutant (FIX-Padua) is used. However, the mechanism by which FIX-Padua works remains ambiguous due to a lack of research models. Here, in situ introduction of *F9-Padua* mutation was performed in human induced pluripotent stem cells (hiPSCs) via CRISPR/Cas9 and single-stranded oligodeoxynucleotides (ssODNs). The hyperactivity of FIX-Padua was confirmed to be 364% of the normal level in edited hiPSCs-derived hepatocytes, providing a reliable model for exploring the mechanism of the hyperactivity of FIX-Padua. Moreover, the *F9* cDNA containing *F9-Padua* was integrated before the *F9* initiation codon by CRISPR/Cas9 in iPSCs from an HB patient (HB-hiPSCs). Integrated HB-hiPSCs after off-target screening were differentiated into hepatocytes. The FIX activity in the supernatant of integrated hepatocytes showed a 4.2-fold increase and reached 63.64% of the normal level, suggesting a universal treatment for HB patients with various mutations in *F9* exons. Overall, our study provides new approaches for the exploration and development of cell-based gene therapy for HB.

## 1. Introduction

Hemophilia B (HB) is an X-linked recessive disorder characterized by coagulation dysfunction, owing to mutations in the *F9* gene and the incurred deficiency of functional coagulation factor IX (FIX). Recurrent bleeding within knees, ankles and elbows resulting from defective FIX is bound up with severe chronic arthritis and death threats [1], which has troubled approximately 200,000 people worldwide each year [2,3]. The most commonly used treatment for HB relies on an alternative therapy, that is, regular infusion of exogenous FIX concentrates for prophylaxis. However, due to the short half-life of FIX, which is only 20 to 24 h in plasma, patients need an infusion every 3 or 4 days. Repeated injections not only produce huge economic costs and infection risks, but also contribute to clotting factor inhibitors that make the treatment ineffective [4].

Fortunately, bleeding manifestations and relevant complications will be relieved as long as the FIX activity is increased to more than 1% of the normal level, meaning that even a small amount of transgene expression of functional FIX shows therapeutic effects [5]. Therefore, gene therapy is fairly suitable for HB. Moreover, the *F9* cDNA, whose length is only 2.8 kb with a 3′ untranslated region (UTR), is easy to load into delivery vehicles, providing favorable prerequisites for genomic editing [6]. Recently, a naturally occurring gain-of-function FIX mutant, designated as FIX-Padua, which was initially identified by Simioni and his colleagues (University of Padua), has been considered for treatment of HB. A single missense mutation of FIX-Padua at the *F9* locus (*F9-Padua*, g.31134G>T, and R338L on the protein level) contributes to an 8-fold increase in the coagulation activity of FIX [7]. Preclinical experiments have shown that viral vectors with the *F9* cDNA containing *F9-Padua* improved the FIX coagulation activity in HB animals for the long haul [8,9,10,11]. Consequently, the research on gene therapy for HB with *F9-Padua* is of great clinical value and application potential.

Nevertheless, it is worth noting that several therapies for hemophilia aimed at accelerating hemostasis by intervening in the clotting cascade have reported unanticipated complications, such as thrombosis and even death [12,13]. It should be a warning that researchers need to fully understand the procoagulant activity and relevant mechanisms of therapeutics. Clinical trials based on FIX-Padua are recruiting a large cohort of subjects, but the biochemical mechanisms by which FIX-Padua exhibits high activity have not been entirely discovered. At present, only Benjamin J. Samelson-Jones et al. demonstrated that instead of an internal activation enhancement, the strengthened interaction of FIX-Padua with its cofactor activated factor VIII (FVIIIa) was fairly critical based on a biochemical platform [14]. A lack of research models that simulate the human phenotype partly accounts for few studies on how FIX-Padua works. The natural occurrence of the *F9-Padua* mutation is extremely novel. So far, only one case has been reported [7], and no cell or animal model containing the *F9-Padua* mutation has been established. A drug Hemgenix depending on FIX-Padua has been approved by the U.S. Food and Drug Administration (FDA) for the treatment of HB [15]. Considering safety implications, it is essential to elucidate the mechanisms of the hyperactivity of FIX-Padua with appropriate research models.

There are many adeno-associated viral (AAV)-based clinical trials for HB [16]. Yet, the presence of antibodies against various serotypes of AAV capsids and the existence of cross-immunity among different serotypes remarkably restrict their widespread application [17]. Beyond this, viral vectors have brought about severe liver damage, such as the sharp increase in alanine aminotransferase [18]. Although AAV transduces hepatocytes, which are the predominant cells that synthesize and secret FIX, the broad tropism and nonspecific entrapment by sinusoidal scavenger wall cells (liver sinusoidal endothelial cells and Kupffer cells) [19,20] also seriously hamper the widespread utility of AAV vectors [21,22]. The safety of viral vectors is still the primary problem, indicating a need for more ideal gene therapy strategies.

Given the conceptual simplicity, specificity, high efficiency, and low toxicity, clustered regularly interspaced short palindromic repeats-associated proteins (CRISPR/Cas) systems have become the most attractive tool for gene engineering and are promising for the treatment of genetic disorders including HB [23,24]. We previously demonstrated that utilizing optimized CRISPR/Cas9, a human *F9* cassette could be ectopically integrated into the ribosomal DNA (rDNA) region of mouse embryonic stem cells ex vivo [25]. After gene transfer, integrated cells going through off-target screening could be selected for subsequent in vivo treatment, reducing unintended risks in the process of genomic editing and adverse reactions. On the other hand, compared with ectopic transgene expression of *F9*, genomic engineering at the *F9* locus seems superior for safety and efficiency, which not only restores target gene function but also retains the intrinsic promoter and regulatory elements. Recent studies have successfully introduced donor DNA repair templates into the *F9* locus of HB mice using CRISPR/Cas9 through tail vein injection, corrected the *F9* gene and achieved hemostasis, confirming that CRISPR/Cas9-mediated therapy is a feasible tactic for the treatment of HB [26]. However, systemic delivery of the donor templates via CRISPR/Cas9 inevitably produces off-target effects [27]. Moreover, it has been reported that at least 3000 different types of aberrant *F9* mutations lead to HB pathology [28], which was not all covered by existing repair templates. Therefore, CRISPR/Cas9-mediated therapeutics for HB still need to be optimized.

To overcome the aforementioned drawbacks, here, we constructed a model of human induced pluripotent stem cells (hiPSCs) containing the *F9-Padua* mutation for the first time by CRISPR/Cas9 combined with single-stranded oligodeoxynucleotides (ssODNs) and verified the elevated activity of FIX-Padua in engineered hiPSCs-derived hepatocytes. The model would provide an available platform for further deep research on the mechanism behind the hyperactivity of FIX-Padua. Moreover, full-length *F9* cDNA containing *F9-Padua* was integrated before the *F9* initiation codon in HB patient derived hiPSCs (HB-hiPSCs). Site-specific integrated clones after off-target screening were differentiated into hepatocytes, successfully secreting FIX with a higher activity, which could be transplanted into HB patients to rescue circulating FIX levels. Our cell-based gene therapy strategy through stem cell engineering would achieve therapeutic effects while minimizing potential risks posed by genomic editing. Theoretically, we provided a universal treatment for HB patients with various mutations in exons of *F9* and were committed to better applying FIX-Padua to gene therapy for HB.

## 2. Results

### 2.1. Introduction of the F9-Padua Mutation into hiPSCs

**Figure 1:**
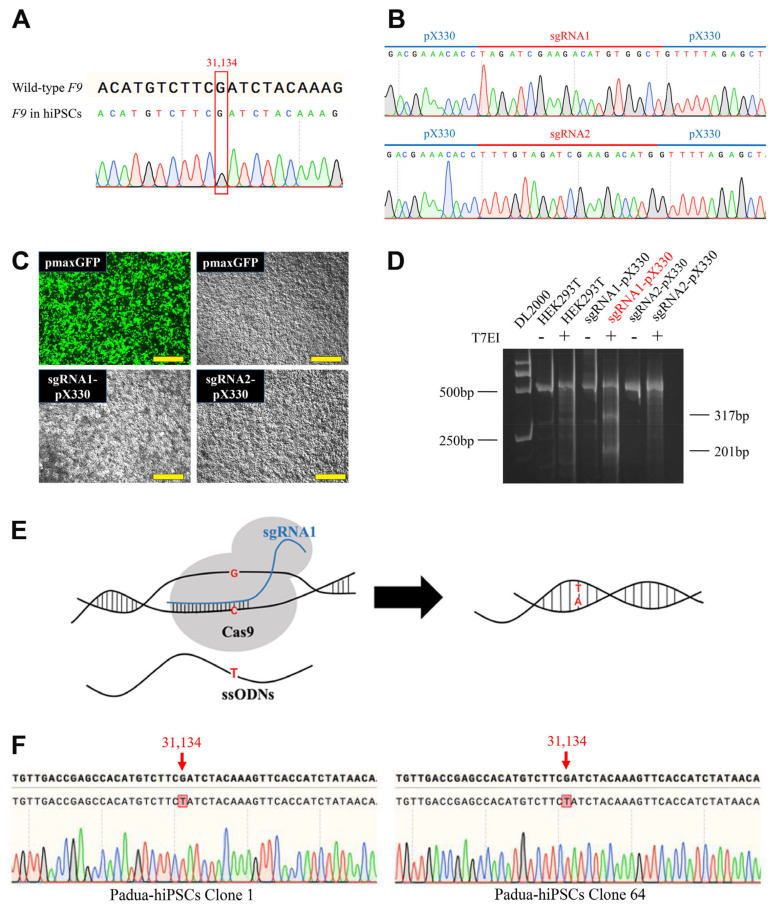
Introduction of the *F9-Padua* mutation into human induced pluripotent stem cells (hiPSCs) derived from normal people. (**A**) Sequencing around site 31,134 of the *F9* gene in hiPSCs. (**B**) Sequencing of sgRNA1-pX330 and sgRNA2-pX330 plasmids. (**C**) Images of human embryonic kidney (HEK293T) cells 24 h after transfected with pmaxGFP, sgRNA1-pX330 and sgRNA2-pX330 vectors, respectively. Green indicates representative cells with green fluorescent protein (GFP). Scale bars, 500 μm. (**D**) Representative gel of T7 Endonuclease I (T7E1) cleavage assay in HEK293T cells for estimating the cutting efficiency of sgRNAs. (**E**) Schematic of introduction of the *F9-Padua* mutation via CRISPR/Cas9 and single-stranded oligodeoxynucleotides (ssODNs). (**F**) Sequencing around site 31,134 of the *F9* gene in 2 positive clones (Padua-hiPSCs) from edited hiPSCs.

The *F9-Padua* mutation seems unique because no other case has been reported in the population after the first proband with thrombophilia resulting from this mutation [29,30]. Before gene editing, to confirm that hiPSCs derived from normal people did not carry the *F9-Padua* mutation, the *F9* gene was amplified with polymerase chain reaction (PCR) and Sanger-sequencing of the PCR products were performed. *F9-Padua* and other mutations were not detected in hiPSCs (Figure 1A). Then, we put the sequence around site 31,134 of the *F9* gene on the sgRNA-designing website CHOPCHOP, after which two highly ranked sgRNAs were picked and inserted into the CRISPR/Cas9 plasmid pX330 (Figure 1B). To choose the more suitable sgRNA for site-directed editing, we transfected sgRNA-pX330 plasmids into human embryonic kidney (HEK293T) cells, with pmaxGFP vectors used as the control for monitoring the transfection efficiency. Twenty-four hours after transfection, cells incorporated with pmaxGFP plasmids showed a strong fluorescence signal, indicating the highly efficient transfection (Figure 1C). For analyzing the cutting efficiency of sgRNAs, cells were collected 72 h post-transfection for T7 Endonuclease I (T7E1) cleavage assay. When editing events occur, 520 bp PCR products amplified with Screen-F/R primers (Table S2) from cells can be cleaved into two small fragments by the T7E1 enzyme. It was obvious that sgRNA1 had undergone satisfactory cutting efficiency, but the cleavage capability of sgRNA2 was significantly weaker (Figure 1D). Hence, sgRNA1-pX330 was chosen for subsequent gene editing in hiPSCs.

The sgRNA1-pX330 plasmid and the 40 bp ssODNs harboring a single-base *F9-Padua* substitution were imported into hiPSCs through nucleofection. SgRNA1-pX330 aimed to snip and break the double-strand DNA at the specific location, inducing DNA damage repair. Additionally, ssODNs containing the *F9-Padua* mutation were applied as templates to introduce the variant g.31134G>T in the genome at the *F9* region through homologous recombination (HDR)-mediated DNA repair (Figure 1E). Upon nucleofection, cells were cultured over a period of time and seeded into single clones. Based on PCR with Screen-F/R primers (Table S2) and Sanger sequencing, 2 out of 107 clones (1.87%, clone 1 and clone 64) were identified as positive clones (Padua-hiPSCs) with successful introduction of the *F9-Padua* point mutation without indels (Figure 1F). Since the hiPSCs used for *F9-Padua* introduction here were obtained from a male (ATCC-DYR0100), clone 1 and clone 64 contained modifications on the single X chromosome.

**Figure 2:**
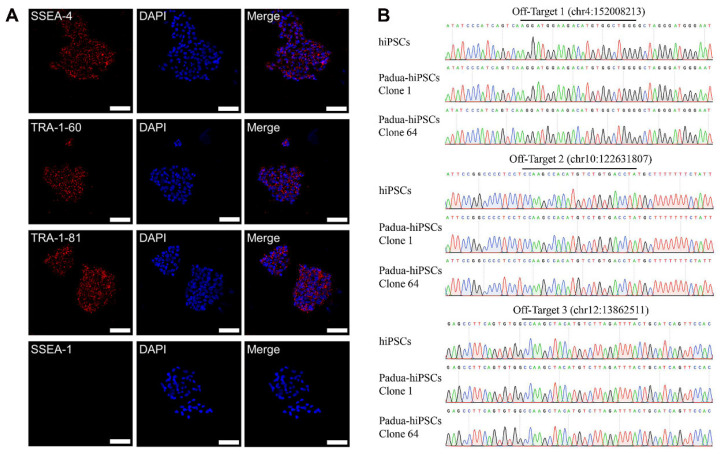
Confirmation of pluripotent characteristics and off-target screening in hiPSCs inserted with the *F9-Padua* mutation (Padua-hiPSCs). (**A**) Immunofluorescence staining with antibodies against stage-specific embryonic antigen 4 (SSEA-4), TRA-1-60, TRA-1-81 and stage-specific embryonic antigen 1 (SSEA-1), as well as 4′-6-diamidino-phenylindole (DAPI) for nuclear indication in Padua-hiPSCs. Scale bars, 75 μm. (**B**) Three potential off-target sites screened in Padua-hiPSCs.

According to immunofluorescence analysis, Padua-hiPSCs expressed typical human pluripotent stem cell markers stage-specific embryonic antigen 4 (SSEA-4), TRA-1-81 and TRA-1-60 (Figure 2A), but did not show stage-specific embryonic antigen 1 (SSEA-1), informing that Padua-hiPSCs remained in an undifferentiated state. To evaluate the off-target risks of the sgRNA1-pX330, we searched for potential off-target sites of sgRNA1 on the website. The prediction suggested no off-target site with one- or two-base mismatches in the genome, and three possible off-target sites with three-base mismatches were listed. Therefore, specific primers (OT1-3-F/R, Table S2) were designed to scrutinize those three sites in Padua-hiPSCs. Precise sequencing results illustrated that Padua-hiPSCs did not generate undesirable off-target modifications (Figure 2B).

### 2.2. Generation and Characterization of HB-hiPSCs

**Figure 3:**
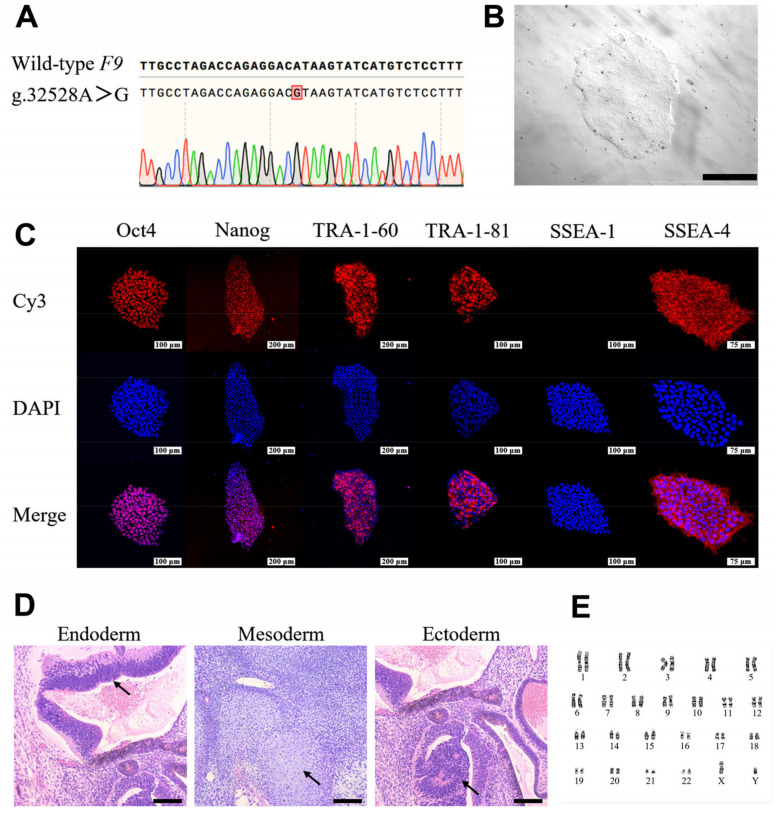
Generation of iPSCs from an HB patient (HB-hiPSCs). (**A**) Identification of the g.32528A>G mutation in the *F9* gene in renal tubular epithelial cells collected from an HB patient. (**B**) Cell morphology of HB-hiPSCs. Scale bar, 500 μm. (**C**) Immunofluorescence staining with antibodies against Oct4, Nanog, TRA-1-60, TRA-1-81, SSEA-1 and SSEA-4, as well as 4′-6-diamidino-phenylindole (DAPI) for nuclear indication in HB-hiPSCs. Secondary antibodies conjugated with Cy3 fluorochromes were used for visualization. Scale bars, 75 μm for SSEA-4; 100 μm for Oct4, TRA-1-81 and SSEA-1; and 200 μm for Nanog and TRA-1-60. (**D**) Hematoxylin and eosin (H&E) staining of teratomas derived from HB-hiPSCs. The arrows indicate representative endodermal, mesodermal and ectodermal tissues, respectively. Scale bars, 200 μm. (**E**) Karyotype analysis of HB-hiPSCs.

We identified an *F9* splicing mutation (g.32528A>G) of a male patient with severe HB (Figure 3A), which was recorded in the Human Gene Mutation Database (HGMD) [31]. Since HB patients are diagnosed with bleeding disorders, renal tubular epithelial cells collected noninvasively from sterile urine rather than skin fibroblasts were used for reprogramming (Figure S1A). According to past investigations, the microRNA-302/367 (miR-302/367) cluster has been reported to enhance the efficiency of iPSCs reprogramming [32,33]. Thereupon, through nucleofection of pEP4EO2SET2K plasmids which carried Oct4, Sox2, SV40 large T and Klf4, along with pCEP4-miR-302/367 plasmids which contained the miR-302/367 cluster, the reprogramming of renal tubular epithelial cells into HB-hiPSCs was conducted. After nucleofection, we observed distinct clones that resembled embryonic stem cells (ESCs) in morphology within 20 days (Figure S1B). Those ESCs-like clones (HB-hiPSCs) were picked and expanded for further characterization and genomic engineering (Figure 3B).

It was demonstrated that cell colonies of HB-hiPSCs were positive for alkaline phosphatase (ALP) staining (Figure S1C). From the images of immunofluorescence, HB-hiPSCs expressed Oct4, Nanog, TRA-1-60, TRA-1-81 and SSEA-4, which were human stem cell markers (Figure 3C). Conversely, SSEA-1 was not detected, consistent with the features of human ESCs. We further characterized the differentiation potential of HB-hiPSCs in vivo by teratoma formation experiments. After being injected subcutaneously into nude mice, HB-hiPSCs formed teratomas with all three germ layers (Figure 3D). To assess the chromosomal normality, karyotyping was performed on HB-hiPSCs. Results revealed the normal karyotype (Figure 3E). It was further validated that after reprogramming, HB-hiPSCs still carried the g.32528A>G mutation in *F9* (Figure S1D), which was used as a model for the following research on HB. To sum up, those results verified the successful generation of HB-hiPSCs.

### 2.3. Integration of Modified Full-Length F9 cDNA with the F9-Padua Mutation in HB-hiPSCs

**Figure 4:**
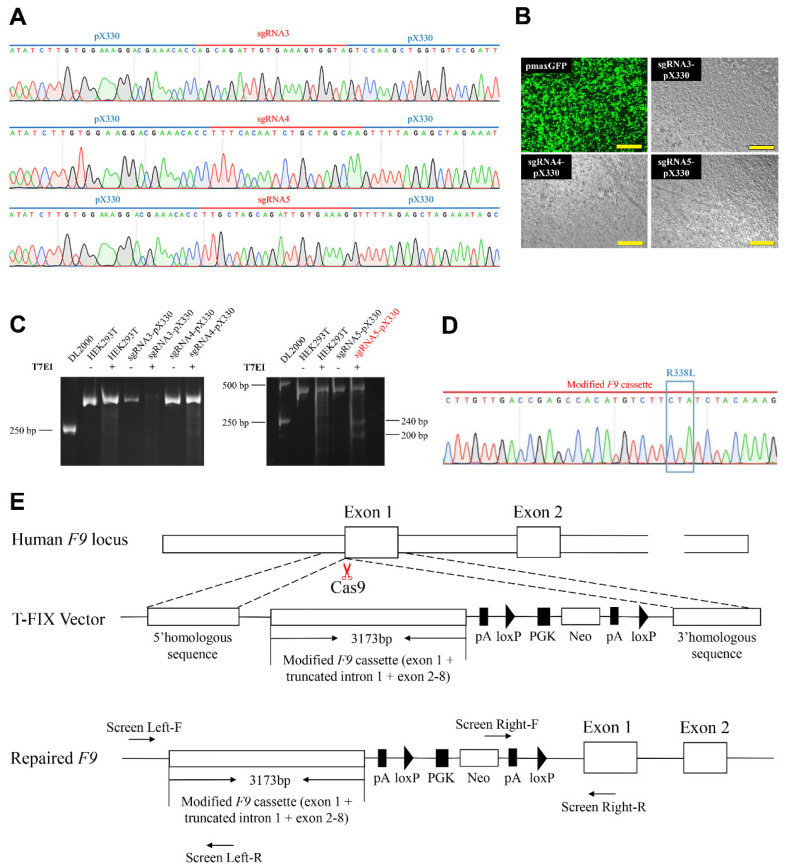
Genomic engineering of *F9* locus before the initiation codon in HB-hiPSCs by CRISPR/Cas9. (**A**) Sequencing of sgRNA3, 4 and 5-pX330 plasmids. (**B**) Images of HEK293T cells 24 h after transfected with pmaxGFP, sgRNA3-pX330, sgRNA4-pX330 and sgRNA5-pX330 vectors, respectively. Green indicates representative cells with green fluorescent protein (GFP). Scale bars, 500 μm. (**C**) Representative gel of T7E1 assay in HEK293T cells for assessing the cleavage efficiency of sgRNA3, 4 and 5. (**D**) Sanger-sequencing for the insertion of full-length *F9* cDNA with the *F9-Padua* mutation (R338L on the protein level) in the T-FIX donor vector. (**E**) Schematic of integrating full-length human *F9* cDNA harboring *F9-Padua* as well as truncated intron 1 into the *F9* locus before the initiation codon in HB-hiPSCs.

Aiming at providing a universal therapeutic regimen for HB, HB-hiPSCs were used for CRISPR/Cas9-mediated FIX restoration by integrating modified *F9* cDNA containing the *F9-Padua* mutation with truncated intron 1 into the *F9* locus before the initiation codon. Three sgRNAs matched the sequence before the *F9* initiation codon were picked from the website CHOPCHOP and inserted into the pX330 plasmid, respectively (Figure 4A). SgRNAs-pX330 plasmids were efficiently delivered into HEK293T cells (Figure 4B) and the cleavage efficiency of each sgRNA was assessed by T7E1 assay (with Screen HB-F/R primers, Table S2). It was shown that sgRNA5 displayed impactful cutting activities and was selected for further experiments, while sgRNA3 and 4 exhibited limited cleavage efficiency (Figure 4C).

**Figure 5:**
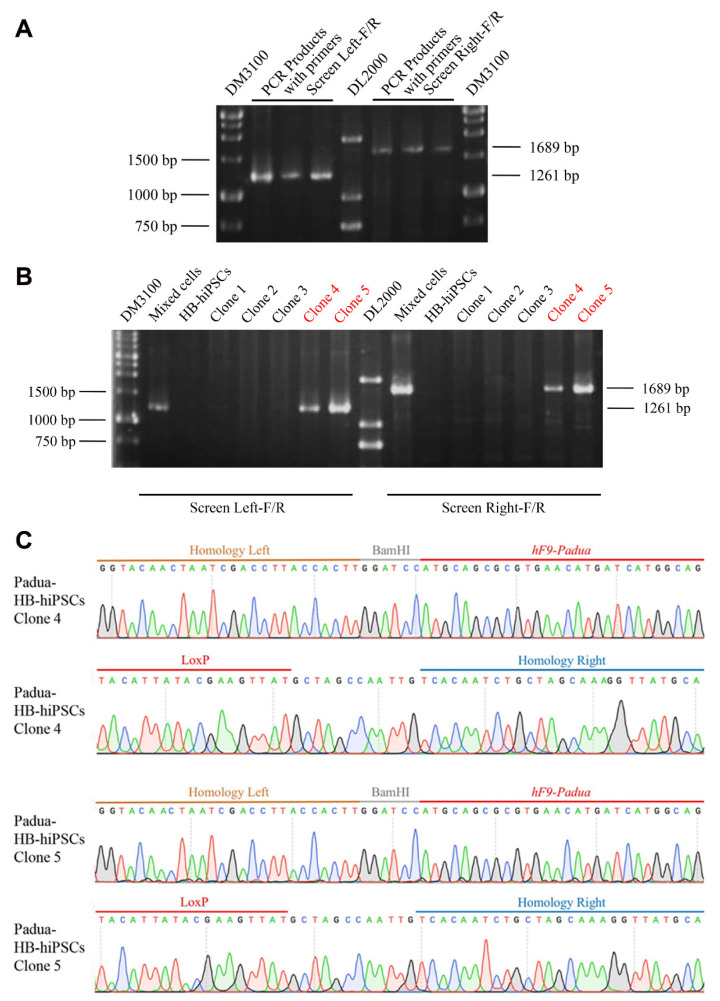
Identification of site-specifically integrated clones from HB-hiPSCs after gene editing. (**A**) PCR amplification with Screen Left-F/R and Screen Right-F/R primers in mixed cells after nucleofection. (**B**) PCR amplification for 5 single clones. (**C**) Sequencing for site-specific integration in 2 positive clones (Padua-HB-hiPSCs).

The sgRNA5-pX330 plasmid and the donor vector T-FIX with full-length *F9* cDNA harboring the *F9-Padua* mutation and truncated intron 1 were guided into HB-hiPSCs by nucleofection (Figure 4D,E). After G418 selection, the drug-resistant mixed cells were subjected to PCR analysis with two paired primers Screen Left-F/R and Screen Right-F/R (Table S2). Mixed cells generated a 1261 bp product and a 1689 bp product, respectively (Figure 5A), which implied the existence of integrated cells in the mixture. Then, single clones were isolated and two out of five clones (40%, clone 4 and clone 5) were recognized as positive clones (Padua-HB-hiPSCs) with site-specific integration and no mutation nearby targeted sequences (Figure 5B,C). 

**Figure 6:**
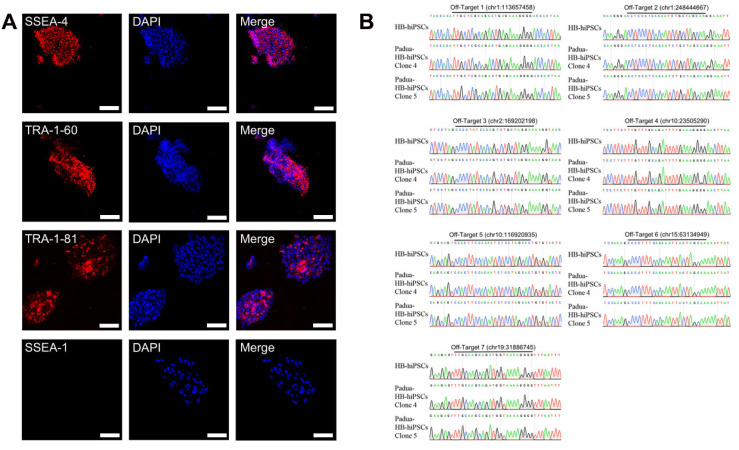
Investigation of pluripotent characteristics and off-target screening in Padua-HB-hiPSCs. (**A**) Immunofluorescence staining with antibodies against SSEA-4, TRA-1-60, TRA-1-81 and SSEA-1 in Padua-HB-hiPSCs. DAPI was used for nuclear staining at the same time. Scale bars, 75 μm. (**B**) Seven risky off-target sites scrutinized in Padua-HB-hiPSCs.

Furthermore, immunofluorescence demonstrated that Padua-HB-hiPSCs maintained the multiple differentiation potential of human pluripotent stem cells, expressing representative human pluripotent stem cell markers SSEA-4, TRA-1-81 and TRA-1-60 but not SSEA-1 (Figure 6A). Similarly, to exclude the off-target cleavage of sgRNA5, a two-base mismatched off-target site and six sites with three-base mismatches predicted from the website were screened in Padua-HB-hiPSCs (with primers OT4-10-F/R, Table S2). As a result, no abnormality was observed around those seven risky sites in the genome (Figure 6B).

### 2.4. Derivation of Hepatocytes from Edited Clones

**Figure 7:**
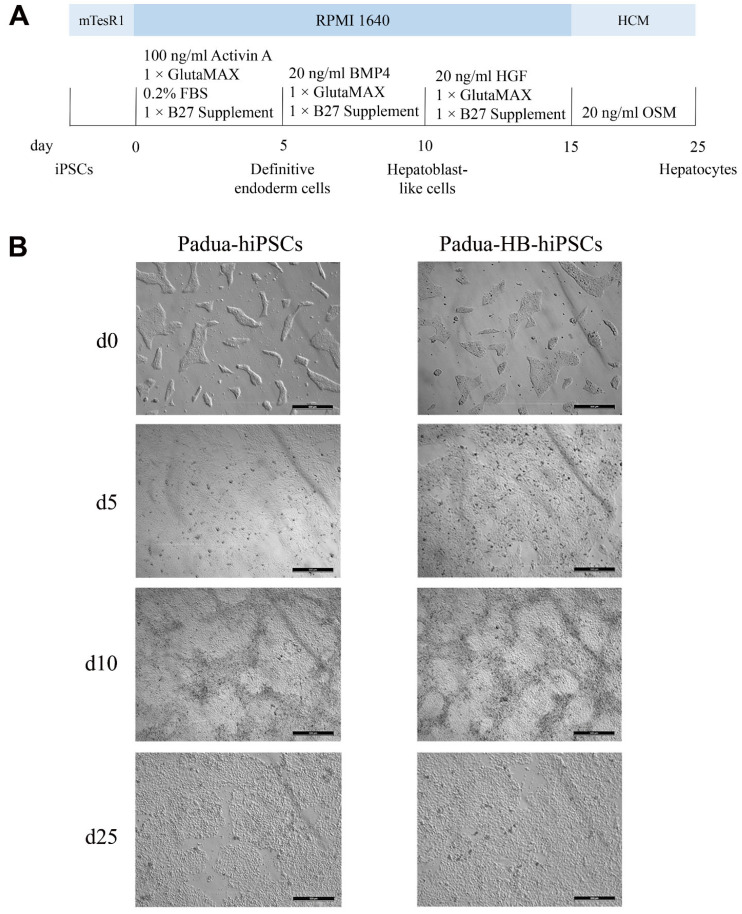
The differentiation of hiPSCs, Padua-hiPSCs, HB-hiPSCs and Padua-HB-hiPSCs into hepatocytes. (**A**) Schematic of the 4-stage differentiation protocol. (**B**) Images of cells captured on day 0, 5, 10 and 25 during the differentiation process. Scale bars, 500 μm.

Given that human circulating FIX was mainly secreted from hepatocytes [34], hiPSCs, Padua-hiPSCs, HB-hiPSCs and Padua-HB-hiPSCs were differentiated into hepatocytes (annotated as hiHCs, Padua-hiHCs, HB-hiHCs and Padua-HB-hiHCs, respectively) following a renewed four-stage protocol (Figure 7A) [35]. During the differentiation course, the cell morphology changed on day 5 from a smooth clone to a spindle or triangular shape, with the feature of endoderm cells appearing, which turned to be polygonal in the next 5 days with distinct graininess (Figure 7B and Figure S2). Ultimately, on day 25, cells were fairly irregularly shaped with the visible nucleus, which exhibited a typical hepatocyte morphology.

**Figure 8:**
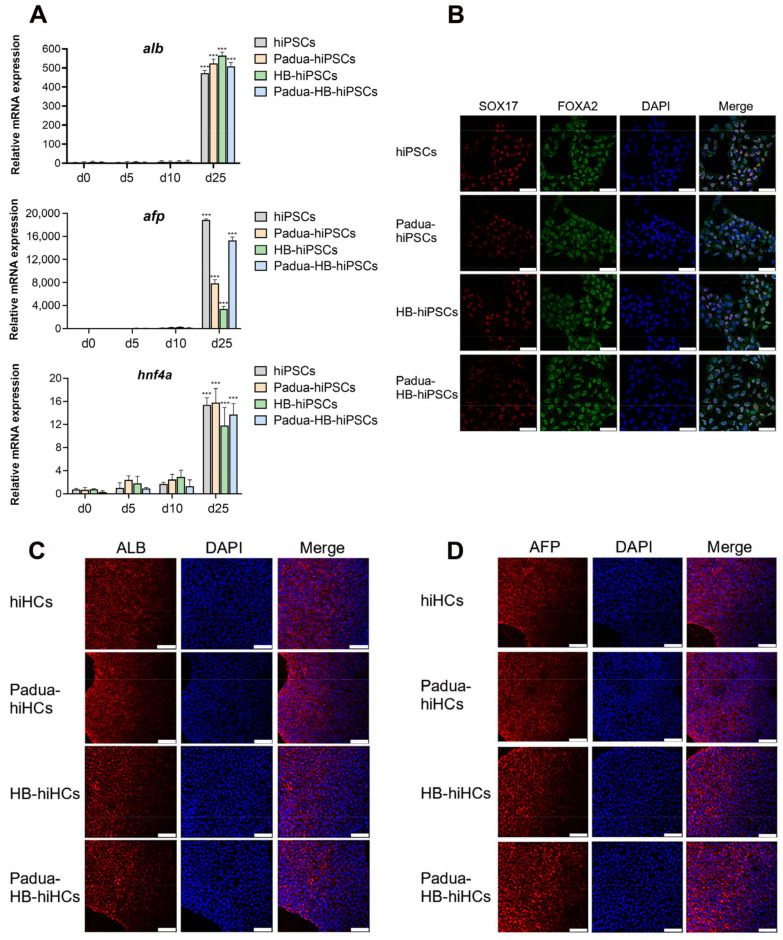
Characterization of differentiated hepatocytes. (**A**) Relative mRNA levels of hepatic markers *ALB*, *AFP* and *HNF4α* (normalized to *HPRT*) in cells differentiated from hiPSCs, Padua-hiPSCs, HB-hiPSCs and Padua-HB-hiPSCs on day 0, 5, 10 and 25 (*n* = 3 for each group). Values on day 5 were set to 1. *** *p* < 0.001. (**B**–**D**) Immunofluorescence staining with antibodies against SOX17, FOXA2, ALB and AFP. DAPI was used for nuclear staining. Scale bars, 75 μm.

To confirm the efficient hepatic differentiation, hepatocyte-specific marker genes were detected with qRT-PCR in cells derived from hiPSCs, Padua-hiPSCs, HB-hiPSCs and Padua-HB-hiPSCs on day 0, 5, 10, and 25. It was illustrated that mRNA levels of *ALB* and *AFP* were dramatically elevated at day 25 in all differentiated cells (Figure 8A). Of note, the differences between the fold changes in *AFP* transcript levels in those four groups of cells may result from the minimally expressed *AFP* gene in the initial stage of differentiation. Additionally, the transcriptional expression of *HNF4α* emerged on the 5th day, which progressively increased in the following 20 days, indicating that cells gradually exhibited hepatocyte characteristics during differentiation. Meanwhile, immunofluorescence staining verified that on the 5th day, differentiated cells were all positive for FOXA2 and SOX17 (Figure 8B), sharing endoderm traits. On day 25, ALB and AFP expressions were distinctly observed in hiHCs, Padua-hiHCs, HB-hiHCs and Padua-HB-hiHCs (Figure 8C,D), which signified the typical hepatocyte phenotype. 

**Figure 9:**
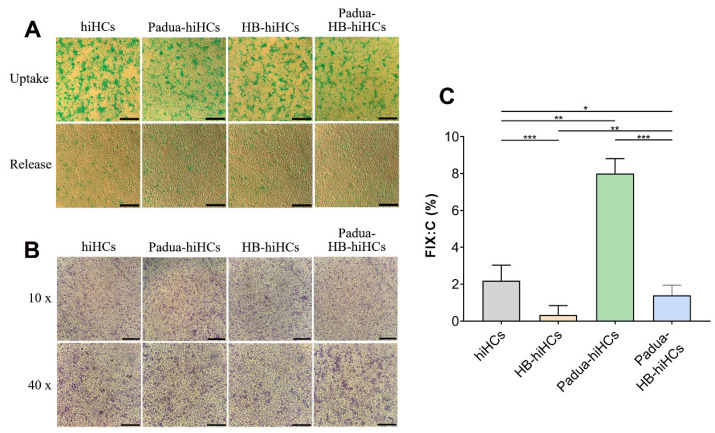
Validation of the hyperactivity of FIX-Padua secreted from edited hepatocytes with mature function. (**A**) Indocyanine green (ICG) uptake analysis on day 25 during the differentiation progress. Green indicates ICG in cells. Scale bars, 200 μm. (**B**) Periodic acid-Schiff (PAS) staining on day 25 observed under an optical microscope at 10 (up) and 40 (down) magnification. Scale bars, 500 μm (up) and 200 μm (down). (**C**) FIX coagulation activity in the supernatant of differentiated hepatocytes (*n* = 3 for each group). * *p* < 0.05; ** *p* < 0.01; *** *p* < 0.001.

Then, the function of differentiated hepatocytes was investigated with an indocyanine green (ICG) uptake assay and periodic acid–Schiff (PAS) staining. As mature hepatocytes phagocytose and release ICG, we exposed the ICG solution to hiHCs, Padua-hiHCs, HB-hiHCs and Padua-HB-hiHCs on the 25th day. Under this condition, ICG was loaded into all cells in an hour (Figure 9A). After being apart from the ICG solution with continuous cultivation for 24 h, the cells gradually released ICG. Further verification based on PAS staining was conducted. The distinct presence of purple-stained glycogen in those cells proved that hiHCs, Padua-hiHCs, HB-hiHCs and Padua-HB-hiHCs gained the function of mature hepatocytes to synthesize and store glycogen (Figure 9B).

Collectively, our results suggested that hiPSCs, Padua-hiPSCs, HB-hiPSCs and Padua-HB-hiPSCs were successfully differentiated into hepatocytes.

### 2.5. Validation of the Hyperactivity of FIX-Padua Proteins Produced by Hepatocytes from the F9-Padua hiPSCs Model and Integrated HB-hiPSCs

We next assessed the FIX coagulation activity in the cell supernatant. The activities of FIX were 2.2 ± 0.84% and 8.0 ± 0.82% in the supernatant of hiHCs and Padua-hiHCs, respectively (Figure 9C). That is, the FIX clotting activity in the culture medium of Padua-hiHCs was approximately 364% of the normal level, with a significant difference, owing to the *F9-Padua* mutation.

In terms of HB-hiHCs, limited FIX activity was detected (0.33 ± 0.52%), which confirmed that the g.32528A>G mutation in the *F9* gene contributed to the deficiency of functional FIX protein production (Figure 9C). By contrast, FIX coagulation activity in the supernatant of Padua-HB-hiHCs was measured to be 1.40 ± 0.55%, showing a 4.2-fold increase compared with HB-hiHCs, and was approximately 63.64% of the normal level.

## 3. Discussion

Targeted genome editing has become one of the most popular topics in the medical field. Researchers constantly attempt to refine nuclease systems that bind to specific DNA sequences and precisely achieve genomic modification. There are three genomic editing tools commonly used: zinc finger nucleases (ZFN), transcription activator-like effector nucleases (TALEN) and CRISPR/Cas systems [36]. ZFN and TALEN have played an important role, but their use is limited by several factors, such as the complex and costly preparation process [37]. Therefore, a more simple, reliable and practical method for genomic editing is needed, promoting the development of CRISPR/Cas systems. Among them, CRISPR/Cas9 is predominantly used. 

For gene therapy, the off-target side effects are not neglectable during the application of CRISPR/Cas9. It has been indicated that sgRNAs are fault-tolerant to some extent, which may trigger mismatches and unexpected editing at nontarget DNA strands [27]. Therefore, we predicted off-target sites that may mismatch with sgRNAs used here, and scrutinized those sites in positive clones. Fortunately, no random insertions or deletions in those predicted loci were observed, indicating that sgRNAs we used had a low off-target risk, which is consistent with the limited incidence of off-target issues in hiPSCs [38,39]. On the other hand, further exploration with whole genome sequencing (WGS) and other sequencing-based methods is warranted for revealing off-target editing events that were undetected by our PCR-based methods.

To date, recombinant FIX with extended half-life and enhanced activity has significantly improved the life quality of patients, yet continuous infusions remain an insurmountable challenge [40]. Apart from recombinant coagulation factor infusions, recent studies to cure HB for whole life through a single treatment have successfully integrated the *F9* cDNA into “safe harbor” sites so that symptoms would be alleviated by endogenous FIX produced from patients [41]. One of the reasons why “safe harbor” locus comes as the therapeutic target is that at least 3000 types of *F9* mutations are reported to be pathogenic [28,42], making it unfeasible to provide the unique repairing regimen in situ for each abnormal genetic phenomenon. By delivering AAV loaded with CRISPR/Cas9 elements, the codon-optimized partial human *F9* gene carrying exon 2–8 and the *F9-Padua* mutation was guided into the intron 1 region of the mouse *Alb* locus, which gained the sustained and effective FIX expression in neonatal and adult HB mice [43]. In addition, *F9* cDNA was inserted into the *AAVS1* locus (located on the intron 1 of *PPP1R12C* on human chromosome 19) in hiPSCs from HB patients by CRISPR system, so that FIX was stably secreted [6]. We previously illustrated that CRISPR/Cas9 helped to efficiently integrate the human *F9* expression cassette into the ribosomal DNA (rDNA) region of mouse embryonic stem cells, enabling gene therapy for HB based on nonviral vectors [25]. 

However, for the ectopic expression of *F9*, exogenous promoters are indispensable. Additionally, the coding sequences of harbor sites may be disrupted and lead to new mutations. In comparison, in situ gene therapy, i.e., performing editing directly in the *F9* locus, is superior in regard to safety and controllability. However, those strategies, such as single-base repair and gene correction for indel variations, are usually suitable for a single type of genetic mutation rather than HB with multiple kinds of pathogenic variants. Facing that obstacle, we presented a more versatile in situ gene therapy scenario for HB.

Given that the FIX-Padua mutant caused by the *F9-Padua* single-base mutation obtains higher coagulation activity than wild-type FIX, which was confirmed in our study, and simultaneously does not lead to thrombotic complications such as hypercoagulability [8], FIX-Padua was considered in this research to achieve better outcomes. Here, we attempted to insert human *F9* cDNA containing truncated intron 1 and exons 1–8 with the *F9-Padua* mutation into the *F9* locus before the initiation codon in the hiPSCs of an HB patient. Hepatocytes derived from site-specifically integrated hiPSCs were observed to secrete FIX stably, with the clotting activity elevated from 0.33 ± 0.52% to 1.40 ± 0.55%. In other words, after gene engineering, the FIX coagulation activity in the supernatant of cells from the patient was significantly enhanced and reached 63.64% of the normal activity level. It was reported elsewhere that when full-length *F9* cDNA was inserted into the *AAVS1* locus of patient-specific hiPSCs, the FIX activity in the supernatant of hepatocytes differentiated from integrated hiPSCs increased from 1.7% to 5% [6]. It is noteworthy that as long as the activity of FIX is increased to more than 1% of the normal level, hemorrhage and relevant complications would be effectively relieved [5]. Hence, our strategy is capable of achieving therapeutic effects, which is worth bringing into clinical application.

To evaluate the mechanism by which FIX-Padua exerts hyperactive activity in the coagulation system, recombinant FIX-Padua proteins and a novel experimental platform where the function of FVIIIa was replaced by emicizumab reagent were used to demonstrate that the high clotting activity of FIX-Padua relied on the enhanced interaction with its cofactor FVIIIa, rather than the activation or inactivation on its own [14]. Researchers also observed a correlation between FVIIIa activity and gene therapy-mediated FIX-Padua coagulation activity in HB subjects. These findings suggest that the molecular regulatory mechanism of FIX-Padua is similar to that of wild-type FIX in physiological conditions. However, so far, no other studies have been reported on the mechanism of the increased activity of FIX-Padua, and the FVIIIa-related principle remains to be confirmed in cells or animals apart from molecular platforms. The hiPSCs model with the *F9-Padua* mutation constructed in this study is of great significance for validating and exploring the mechanism of the hyperactivity of FIX-Padua from broader perspectives. It provides more extensive basic data for subsequent clinical trials and offer solid foundations for the safety and practicality of gene therapy for HB.

Moreover, based on cell models, other gain-of-function variants of FIX with improved activity were gradually identified [44,45]. A FIX-triple mutant with three substitutions (V86A/E277A/R338A) was modified whose activity was 13-fold higher than wild-type FIX after protein purification and was 3.5-fold higher in HB animals [46]. Further optimization of that variant, with R338A being replaced by R338L, generated the FIX-tripleL. FIX-tripleL showed 22-fold higher activity than wild-type FIX as recombinant proteins and exhibited 15-fold stronger activity in vivo, which seems better than FIX-Padua [47]. FIX-E456H and FIX-R384L mutants were also reported to achieve a 7- and 8.6-times improvement in clotting activity, respectively [48].

It can be seen that more effective FIX mutants are generated in addition to FIX-Padua, in the hope of better treating HB with lower injection dosages and immunological responses. Considering that excessive coagulation activities contribute to unexpected thrombosis, FIX-Padua still has the greatest potential for clinical treatment. When the *F9-Padua* mutation was inserted in hiPSCs using CRISPR/Cas9 combined with ssODN, it was confirmed that in this cell-model-derived hepatocyte, FIX-Padua demonstrated 3.64-fold higher activity than wild-type FIX. However, it was recorded that the activity of FIX-Padua in the plasma of the thrombosis patient was 776% of the normal level, and in vitro experimental results showed that the activity of FIX-Padua was up to eight times the activity of wild-type FIX [7], which was much higher than we measured. It is probably because HEK293 cells were used for FIX level detection in previously published research, whereas coagulation factors were mainly secreted by hepatocytes. In this study, hepatocytes derived from hiPSCs were selected to compare the activity of wild-type FIX and FIX-Padua. The characteristics and functions of organisms are more likely to be preserved in hiPSCs than in other cell lines [49,50]. Consequently, our results are more closely attuned to the actual analysis. Furthermore, FIX-Padua activity varies dramatically with the discrepancies in assays used for FIX coagulation activity measurement in different institutions [51]. 

Currently, most preclinical and clinical trials for the gene therapy of HB focus on the delivery of viral vectors, with a preference for AAV vectors, to obtain the expression of exogenous *F9* cDNA [52]. In this way, the concentrations of circulated FIX in the plasma of treated animals or patients are significantly increased and maintained for a certain period [53]. It was detected that the FIX activity in mouse plasma was stabilized at 147.3% ± 21.0% within 24 weeks after the engraftment by AAV vectors and CRISPR/Cas9 [43], which is therapeutic and meaningful. Yet, there are still concerns about the safety of viral vector-based delivery. Undesired side effects should be addressed, including the immune response, toxicity, germline transmission and tumorigenesis. In particular, random integrations may disrupt open reading frames (ORFs) or cis-activating regulatory elements, leading to uncontrolled mutagenesis and genotoxicity [54]. Moreover, the insertion of strong promoters may activate adjacent genes, including oncogenes. In the absence of a preserved signal or silencer, potential proteome instability accompanied by the overexpression of therapeutic cassettes may also induce the transient expression of neighboring genes and the activation of oncogenes or disruption of functional gene frames [55]. Of note, therapeutic viral vectors are prone to be lost along with the cell cycle progression. Thereafter, the continuous transgene expression is hindered [56]. The broad tropism and nonspecific entrapment by nontargeted cells severely limited the widespread utility of AAV vectors as well [21,22].

In this regard, cell-based gene transfer strategies would minimize those downsides. Site-specific integrations and side effects can be detected during cell culturing ex vivo to rule out clones that may lead to catastrophic outcomes. Transplantation of those selected cells would overcome hurdles brought out by viral vectors [57]. Considering that hiPSCs maintain the self-renewal ability and the multiple differentiation potential similar to embryonic stem cells, but without ethical issues, they bring forward personalized cell therapy, disease modeling and drug screening towards a new platform. Hepatocytes derived from iPSCs were intrasplenically transplanted into hemophilia B mice, enhancing the FIX clotting activity and reducing the time of thrombus generation [58]. Furthermore, transplanting iPSC-derived hepatocytes under the kidney capsule also obtained significant therapeutic effects in FIX-deficient mice [59]. We successfully engineered hiPSCs, which could be differentiated into hepatocytes or other cell types with therapeutic levels of FIX. Additionally, therapeutic cells can be infused back into HB patients, thus avoiding adverse reactions such as off-target effects caused by the direct injection of viral vectors. Remarkably, hiPSCs used for gene editing can be generated from patients with noninvasive methods. For example, urine epithelial cells could be collected and reprogrammed into hiPSCs. Then, autologous cell therapy would be conducted simultaneously to limit the occurrence of immunological rejection. On the other hand, undifferentiated hiPSCs may still form teratomas and come with safety concerns, so strategies (such as fluorescence-activated cell sorting) to purify hepatocytes and remove undifferentiated hiPSCs are supposed to be considered before transplantation.

## 4. Materials and Methods

### 4.1. Plasmid Construction

SgRNAs were designed on the website (http://chopchop.cbu.uib.no, accessed on 1 September 2020) and inserted into the pX330 plasmid (generously provided from Feng Zhang). The sgRNA1 and 2, matching sequences around site 31,134 of the human *F9* gene, were candidates for *F9-Padua* introduction in hiPSCs derived from normal people, while sgRNA3, 4 and 5, targeting sequences before the initiation codon of *F9*, were selected for HB-hiPSCs engineering. The sgRNAs used were listed in Table S1. The 40 bp ssODNs were homologous with sequences around site 31,134 of the human *F9* gene, with the base G substituted by T at site 31,134 (*F9-Padua*), and were synthesized by bio companies (Sangon Biotech, Shanghai, China). Modified full-length human *F9* cDNA containing *F9-Padua* and truncated intron 1 was intended to be integrated into HB-hiPSCs before the initiation codon of *F9*. So, this *F9* cassette, *Neo* coding frames and homologous arms were amplified with the PCR method, respectively, and ligated to pGEM-T Easy Vector (Promega, Madison, WI, USA) with the help of ClonExpress Ultra One Step Cloning Kit (Vazyme Biotech Co., Nanjing, China) in order to construct the donor plasmid T-FIX. 

### 4.2. Generation of HB-hiPSCs from Renal Tubular Epithelial Cells

All subjects gave their informed consent for inclusion before they participated in the study. The study was conducted in accordance with the Declaration of Helsinki, and the protocol was approved by the Ethics Committee of the Center for Medical Genetics of Central South University (No. 2021-1-40, approval date: 1 July 2021). Using the phenol–chloroform method [60], the genomic DNA (gDNA) was extracted from renal tubular epithelial cells collected from the sterile urine of normal people and an HB patient, respectively. Human *F9* gene was amplified from the gDNA of normal people and the HB patient with primers Screen-HB-F/R (Table S2). Sanger sequencing of PCR products was performed. The miR-302/367 cluster was amplified by PCR from the gDNA of normal people with primers HSA-F/R (Table S2) and cloned into pCEP4 plasmids (Addgene, Cambridge, MA, USA). Next, renal tubular epithelial cells from the HB patient were reprogrammed through the nucleofection of pEP4EO2SET2K plasmids (Addgene, Cambridge, MA, USA) and pCEP4-miR-302/367 plasmids with the Nucleofector instrument (Lonza, Basel, Switzerland) and the Amaxa Human Stem Cell Nucleofector Starter Kit (Lonza, Basel, Switzerland). After a period of cultivation (8–20 days), embryonic stem cell (ESC)-like clones were picked and seeded into Matrigel-coated plates for expansion and characterization. 

### 4.3. Alkaline Phosphatase (ALP) Staining

In 12-well plates, HB-hiPSCs were tested with ALP staining. Briefly, after washed by phosphate-buffered saline (PBS; Gibco, Grand Island, NY, USA) and fixed in 4% paraformaldehyde (PFA) solution for 1 min, cells were stained using the ALP detection kit (Beyotime Biotechnology, Shanghai, China) according to the instructions. Images were captured with a camera (Leica Microsystems, Wetzlar, Germany).

### 4.4. Karyotyping

When the cell confluence reached 60%, colchicine (Sigma-Aldrich, Burlington, MA, USA) was added into the culture medium. Five hours later, cells were collected and centrifuged at 1500 rpm for 10 min. After being resuspended in 0.075 mol/L potassium chloride (KCl) solution, the cells were incubated at 37 °C for 20 min, immobilized by Carnoy fixative and dried on clean glass slides. G-bands of chromosomes were visualized using trypsin treatment and Giemsa staining (Sigma-Aldrich, Burlington, MA, USA).

### 4.5. Teratoma Formation Experiments

Animals were housed at the animal facility in the Center for Medical Genetics of Central South University. The care and use of the animals complied with the guidelines of the Ethics Committee of the School of Life Sciences of Central South University. All animal experiments were conducted with the approval of the Institutional Animal Care and Use Committee of the Center for Medical Genetics of Central South University. A total of 2 × 10^6^ HB-hiPSCs were injected into BALB/c nude mice (male, 4-week-old) subcutaneously. For this procedure, 3 mice were used. After 6 weeks, the mice were euthanized with Nembutal (Ovation Pharmaceuticals, Inc., Deerfield, IL, USA). Teratomas were sliced and observed with hematoxylin and eosin (H&E) staining (Sigma-Aldrich, Burlington, MA, USA). Photographs were taken under a microscope (Leica Microsystems, Wetzlar, Germany).

### 4.6. Cell Culture

Human embryonic kidney (HEK293T) cells and hiPSCs (DYR0100, obtained from a male) were purchased from ATCC. HB-hiPSCs were generated by us previously. HEK293T cells were cultured with high-glucose Dulbecco’s Modified Eagle’s Medium (DMEM, Gibco, Grand Island, NY, USA) containing 10% FBS (Gibco, Grand Island, NY, USA). HiPSCs and HB-hiPSCs were maintained on Matrigel (Corning, Lowell, MA, USA) with mTeSR1 Plus medium (Stemcell Technologies, Cambridge, MA, USA). The medium was changed every day and cells were passaged when 80% confluence was reached.

### 4.7. HEK293T Transfection

The sgRNA-pX330 plasmids were transfected, respectively, in HEK293T cells using jetPRIME transfection reagent (Polyplus Transfection, San Marcos, CA, USA), with the pmaxGFP vector used as the control for transfection efficiency. The medium was changed 2 h before transfection. Amounts of 200 μL of transfection buffer, 2 μg of individual plasmid, and 8 μL of jetPRIME reagent were mixed well, set aside for 15 min, and added to cells in six-well plates. The medium was refreshed 12 h later and changed every other day. Cells were observed under a fluorescent microscope (Leica Microsystems, Wetzlar, Germany) 24 h after transfection and harvested for T7E1 investigation 72 h after plasmid delivery.

### 4.8. T7E1 Cleavage Assay

The genomic DNA was extracted from cells using the phenol–chloroform method previously described [60]. Sequences around the cutting sites of sgRNAs were amplified, respectively, by PCR using specific primers (Table S2). The PCR products were annealed in NEB buffer 2 (New England Biolabs, Ipswich, MA, USA), and incubated with T7E1 enzyme (Vazyme Biotech Co., Nanjing, China) at 37 °C for 55 min, followed by the polyacrylamide gel electrophoresis.

### 4.9. hiPSCs Nucleofection and Genotyping

The medium was refreshed 2 h before nucleofection. Using the Nucleofector instrument (Lonza, Basel, Switzerland) and the Amaxa Human Stem Cell Nucleofector Starter Kit (Lonza, Basel, Switzerland), hiPSCs derived from normal people in 12-well plates were electroporated with 5 μg of sgRNA1-pX330 plasmid and 50 pmol of ssODNs, while HB-hiPSCs were electroporated with 5 μg of sgRNA5-pX330 plasmid and 5 μg of T-FIX plasmid. Upon nucleofection, cells were cultured for 12 h with mTeSR1 Plus medium supplemented with 10 μM Y27632, after which the medium was changed every day. Single cells were seeded into 6 cm dishes coated with Matrigel and continuously expanded. In particular, HB-hiPSCs were screened with 50 μg/mL G418 during this process. After 15 days, single clones were picked by sterile pipette tips to 48-well plates with one clone per well. The genomic DNA was extracted from individual clones and identified using PCR method (with primers in Table S2).

### 4.10. Off-Target Analysis

Potential off-target sites of sgRNA1 and sgRNA5 were predicted on the website (http://chopchop.cbu.uib.no, accessed on 1 September 2020). Thereafter, primers (Table S2) were designed to cover those highly ranked off-target sites. PCR was performed and products were Sanger-sequenced by bio companies (Sangon Biotech, Shanghai, China). 

### 4.11. Immunofluorescence Analysis

Cells were seeded on sterile glass sheets coated with Matrigel. When the confluence reached 60%, PBS was used to wash the cells 3 times. After immobilized in 4% PFA solution, cells were permeabilized by PBS containing 0.1% Triton X-100 for 15 min and blocked in 5% bovine serum albumin (BSA) solution for another 30 min. All antibodies used were purchased from Abcam (Cambridge, MA, USA). Primary antibodies (1:300), fluorescein-conjugated secondary antibodies (1:200) and 4′-6-diamidino-phenylindole (DAPI) were added, respectively. Then, glass sheets were placed on slides, fixed by glycerin and observed with a confocal microscope (Leica Microsystems, Wetzlar, Germany).

### 4.12. Hepatocyte Differentiation

According to the following step, hiPSCs were differentiated into hepatocytes (Figure 7A) [35]. At first, mTeSR1 Plus was changed to RPMI 1640 medium (Gibco, Grand Island, NY, USA) added with GlutaMax (Gibco, Grand Island, NY, USA), 100 ng/mL human Activin A (Biolegend, San Diego, CA, USA), 0.2% FBS and B27 Supplement (Gibco, Grand Island, NY, USA). Five days later, cells were maintained in RPMI 1640 medium with 20 ng/mL BMP4 (Biolegend, San Diego, CA, USA), GlutaMax and B27 Supplement for another 5 days. Afterwards, the medium was replaced by RPMI 1640 containing 20 ng/mL HGF (Biolegend, San Diego, CA, USA), GlutaMax and B27 Supplement for the next 5 days. In the final stage, hepatocyte culture medium (HCM; Lonza, Basel, Switzerland) supplemented with 20 ng/mL OSM (Peprotech, Rocky Hill, NJ, USA) was used for continuous cultivation for at least 10 days.

### 4.13. qRT-PCR Analysis 

Total RNA was extracted from cells with TRIzol Reagent (Invitrogen Life Technologies, Carlsbad, CA, USA) and the phenol–chloroform method [61]. HiScript II Q RT SuperMix for qPCR (+gDNA wiper) kit (Vazyme Biotech Co., Nanjing, China) was used for cDNA synthesize. After the dilution of cDNA, relative gene expressions were evaluated with paired primers (Table S2) using ChamQ SYBR qPCR Master Mix (Vazyme Biotech Co., Nanjing, China) on the CFX Real-Time PCR Detection System (Bio-Rad Laboratories, Hercules, CA, USA). The *HPRT* gene was chosen for standardization.

### 4.14. ICG Uptake Assay

Hepatocytes were washed by PBS for 3 times, added with 50 μM ICG solution (MedChemExpress, Shanghai, China) and cultured at 37 °C for 1 h. Images were captured at that time. Then, cells were incubated in HCM medium supplemented with 20 ng/mL OSM for 24 h and observed again.

### 4.15. PAS Staining

PAS staining was carried out using the commercial kit (Solarbio, Beijing, China) in accordance with the instructions. To be more specific, hepatocytes were immobilized first with the given fixative solution for 15 min. After 3-times PBS washing, the oxidizing agent was added and incubated for 15 min. Then, cells were washed and dyed for 15 min with Schiff Reagent out of the light. The dyeing solution was scoured off by sodium sulfite solution. In the end, cells were stained with hematoxylin followed by visualization.

### 4.16. FIX Coagulation Activity Test

The cell culture medium was collected and centrifuged at 3000 rpm for 5 min. After centrifugation, the supernatant was carefully transferred into new tubes and sent to Kingmed Diagnostics (Changsha, China), performing the one-stage FIX coagulation activity (FIX:C) detection.

### 4.17. Statistical Analysis

SPSS software version 23 (Chicago, IL, USA) was used to perform statistical tests. Data were analyzed with the unpaired Student’s *t* test between 2 groups and *p* values < 0.05 were considered significant. Results are shown as means ± SEM.

## 5. Conclusions

In summary, this study not only presented the cell model of hiPSCs and hiPSCs-derived hepatocytes incorporated with the *F9-Padua* mutation, but also achieved the recovery of FIX activity in hepatocytes derived from the hiPSCs of an HB patient through CRISPR/Cas9, with a universal gene therapy regimen established for HB patients with various mutations in *F9* exons. Our work provides a proof of concept for applying cell-delivering FIX-Padua to treating HB. We offer new approaches for utilizing CRISPR/Cas9 in treating genetic diseases and pave the way for the clinical treatment of HB.

## Data Availability

The data supporting the findings of this paper are presented in the article including Supplementary Materials and are available from the corresponding author upon reasonable request.

## Supplementary Materials

The supporting information can be downloaded at: https://www.mdpi.com/article/10.3390/ijms24109013/s1.
