# Supplementary material for: CRISPR-Mediated In Situ Introduction or Integration of F9-Padua in Human iPSCs for Gene Therapy of Hemophilia B
Source: Int J Mol Sci. 2023 May 19;24(10):9013. doi: 10.3390/ijms24109013 (PMC10219373; doi:10.3390/ijms24109013)
Supplement: Supplementary file 1 [file ijms-24-09013-s001.zip › Figure S1.pdf]

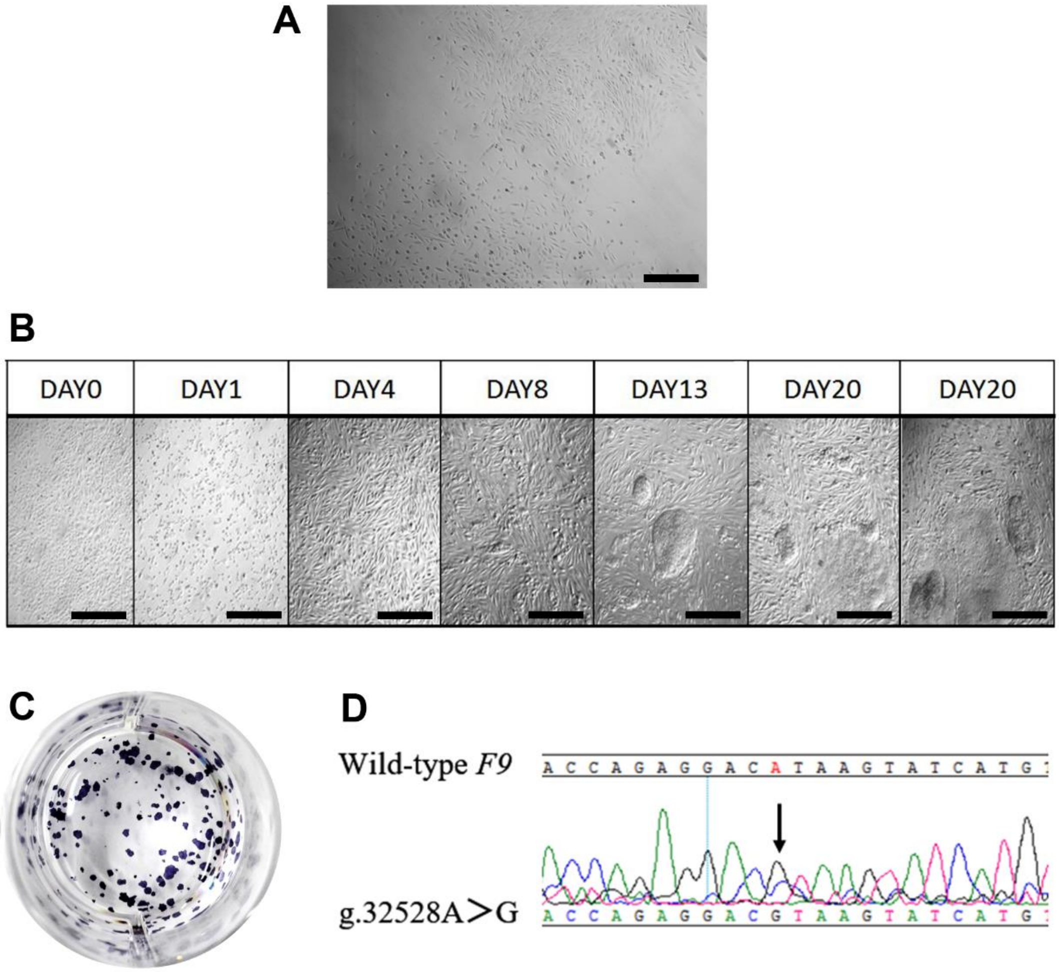

Figure S1. Reprogramming of patient-derived renal tubular epithelial cells into HB-hiPSCs. (A). Cell morphology of primary renal tubular epithelial cells collected from an HB patient. Scale bar, 200 μm. (B). Cell morphology during the reprogramming process from day 0 (DAY0) to day 20 (DAY20). Scale bars, 200 μm. (C). Alkaline phosphatase (ALP) staining of HB-hiPSCs. (D). Verification of the g.32528A > G mutation in the *F9* gene in HB-hiPSCs.
