# Supplementary material for: CRISPR-Mediated In Situ Introduction or Integration of F9-Padua in Human iPSCs for Gene Therapy of Hemophilia B
Source: Int J Mol Sci. 2023 May 19;24(10):9013. doi: 10.3390/ijms24109013 (PMC10219373; doi:10.3390/ijms24109013)
Supplement: Supplementary file 1 [file ijms-24-09013-s001.zip › Figure S2.pdf]

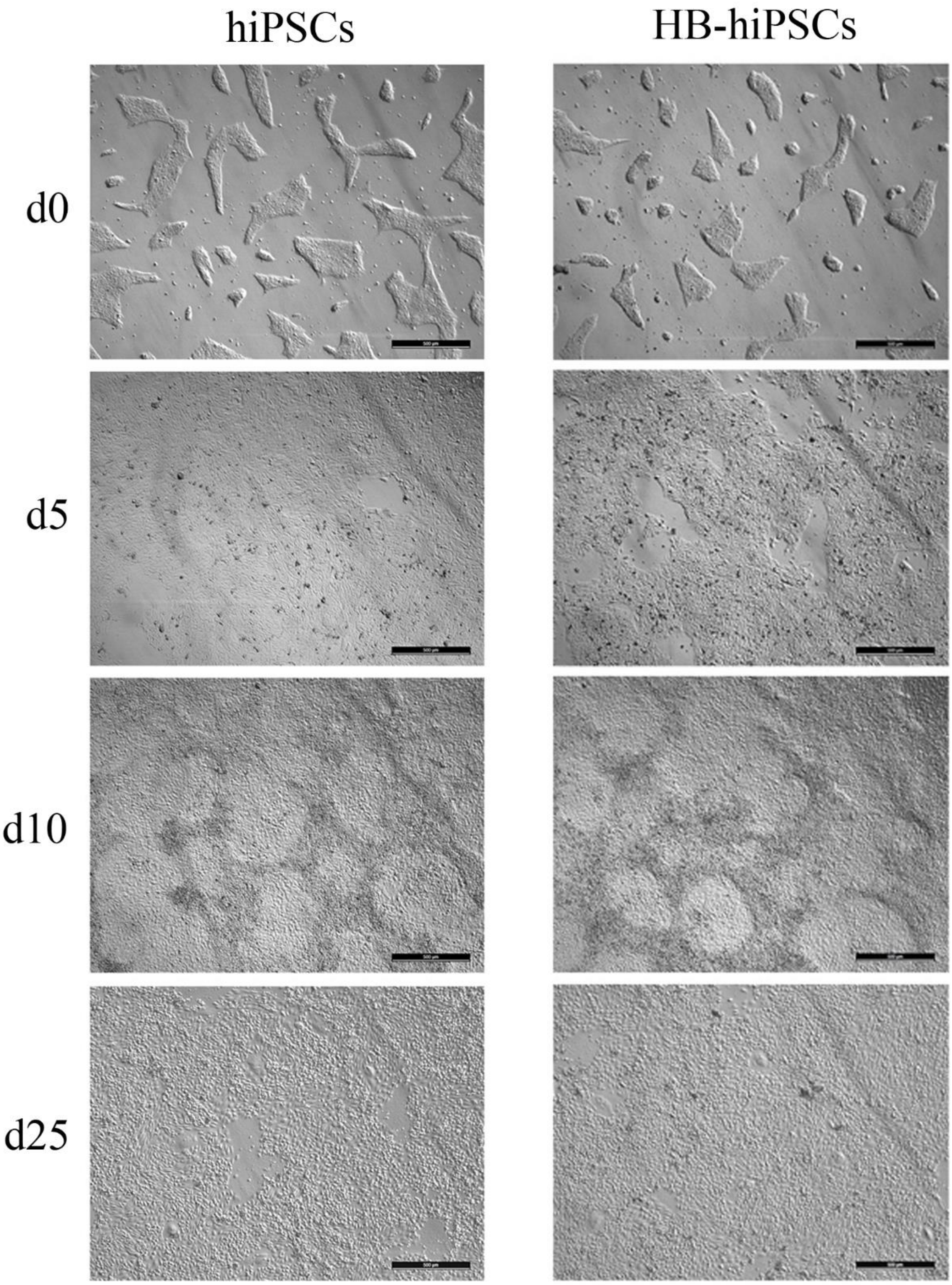

Figure S2. Images of cells captured on day 0, 5, 10 and 25 during the hepatic differentiation process. Scale bars, 500 μm.
