# Supplementary material for: CRISPR-Mediated In Situ Introduction or Integration of F9-Padua in Human iPSCs for Gene Therapy of Hemophilia B
Source: Int J Mol Sci. 2023 May 19;24(10):9013. doi: 10.3390/ijms24109013 (PMC10219373; doi:10.3390/ijms24109013)
Supplement: Supplementary file 1 [file ijms-24-09013-s001.zip › Table S1.pdf]

Table S1. SgRNAs designed on the website

Name	Sequences
sgRNA1	TAGATCGAAGACATGTGGCT
sgRNA2	TTTGTAGATCGAAGACATG
sgRNA3	AGCAGATTGTGAAAGTGGTA
sgRNA4	TTTCACAATCTGCTAGCAA
sgRNA5	TTGCTAGCAGATTGTGAAAG
