# Supplementary material for: CRISPR-Mediated In Situ Introduction or Integration of F9-Padua in Human iPSCs for Gene Therapy of Hemophilia B
Source: Int J Mol Sci. 2023 May 19;24(10):9013. doi: 10.3390/ijms24109013 (PMC10219373; doi:10.3390/ijms24109013)
Supplement: Supplementary file 1 [file ijms-24-09013-s001.zip › Table S2.pdf]

Table S2. Primers used in this paper

Name	Forward (5'-3')	Reverse (5'-3')
Screen	CAACCATGACATTGCCCTTCTGG	GTGATTAGTTAGTGAGAGGCCCTG
Screen-HB	CAGACTCAAATCAGCCACAGTGG	CGTGCTGGCTGTTAGACTCTTC
HSA	CTAGCTAGCGAACACGAATCTTTGGGAAC	GTTCCCAAAGATTCGTGTTCGCTAGCTAG
Screen Left	GCCATTCAATCGAGGAAGGATAGG	CTGAATTATACCTCTTTGGCCGATTC
Screen Right	CAATGCGGCGGCTGCATA	CCCCTAGGGAACTCAACTGAGTAT
OT1	CACATTTGTTTCTCACAGGTCTGTG	TTACTACATGGCTCTGTCCGATTGT
OT2	GGGTGTTATGACCTCAGCTGTAAT	GCACACTATCCTGGTGGCC
OT3	CCCCAAATCTCAGAGAAAAAAGCCT	CAAAAGTCTTCAGAAGAATGCTGTCCT
OT4	ATGTCTCCTTTATTCCTACTGAGGTCAAT	GTCAAATATGCATTTGTTTCAGGGGAG
OT5	CCTGAAAAGAAATGTTTTAAGAGCACTAAAG	GGCCCTGTGGTGACCTTCTA
OT6	CATGGTGCAAGCTAAGTCTAGCTTA	GCAAAATGGACTGCATGCAAAG
OT7	CATGGTGTTTTTCACTGATTATCTTGC	GAGGCATAAACTACTATATTCAATTGTCTGG
OT8	CTTAGTATGTACTACTCCACTGTTGTGT	CCTACCTATTAGACCTCCTCTCTTGG
OT9	AGCCATCTATGCCTCTGGC	GCAAGGTAAAAGTAATAGTTGATTTGTTAAATGT
OT10	CCCCTGCAATCATTTGTTTCAGAT	GTATTTTATGTAGAACTGCCTGCTTTTTATG
ALB	CGCTATTAGTTCGTTACACCA	TTTACAACATTTGCTGCCCA
AFP	TGGGACCCGAACTTTCCA	GGCCACATCCAGGACTAGTTTC
HNF4α	CCAAGTACATCCCAGCTTTC	TTGGCATCTGGGTCAAAG
HPRT	GACTTTGCTTTCCTTGGTCAGG	AGTCTGGCTTATATCCAACACTTCG
